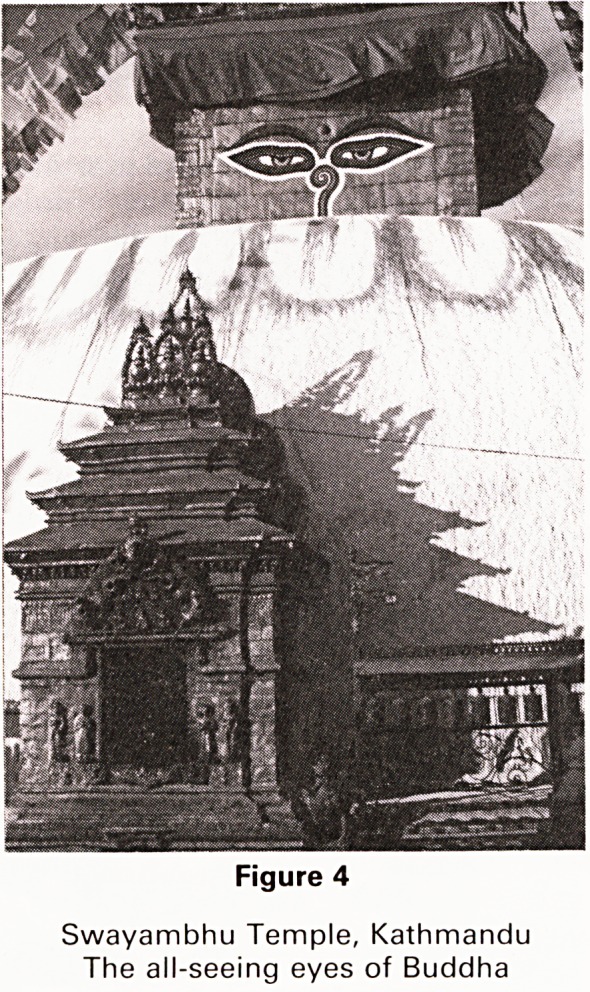# From Our Foreign Correspondent

**Published:** 1986-12

**Authors:** J. L. Burton


### From Our Foreign Correspondent

### 'Bus 237 to Pokhara

J L Burton

Tiring of the touts and tourists in Kathmandu, I decided to travel to Pokhara to see the sun rise over Annapurna. In order to experience the rural way of life of the Nepalese, I scorned the comfortable tourist-bus in favour of the service-bus used by the peasants. At £1.30 for a journey of 130 miles it seemed a bargain—but subsequent experience proved otherwise.

Kathmandu bus-station is a mechanized Bedlam in a dust-bowl. It resembles a huge gypsy encampment, with serried ranks of grimy single-decker buses in place of caravans. Dozens of revving engines drown the shouts of peasants loading and unloading huge bundles from the roof-racks. Goats, dogs and hens add to the din, and vendors cry their wares, which consist mainly of unidentifiable morsels fried in sizzling batter over camp-fires, to be washed down with dirty glassfuls of warm white curds. Scattered heaps of garbage and the occasional dead dog add a touch of local colour, or its olfactory equivalent.

After wandering through a thick haze of dust, exhaust fumes, and wood-smoke, I eventually found a row of ticket-booths, each with its destination-board labelled in Nepali script. Even years of deciphering the hieroglyphics of my clinical assistants did not enable me to identify one that said Pokhara, and I had to stumble along like a blind beggar shouting 'Pokhara? Pokhara?' until some kind soul led me to the right queue. Finding the right bus was even trickier, since my ticket simply said 'Bus 237, seat 47'. More helpless pantomime and at length a ragged barefoot urchin grabbed my ticket, led me through the maze of buses to No. 237, marched me down the aisle and with a proud proprietorial flourish showed me seat 47, which was to be my home for the next 10 hours. My guide, who looked about 8 years old and smoked like a chimney, had clearly never captured a European of any size before, and until our departure he kept returning at intervals to exhibit me to small parties of his friends.

Over the next half hour the bus slowly filled up with large numbers of the Nepalese lower orders, and when it was full to capacity, about 30 more were squeezed in, and we were off.

**Figure 1**

Sunrise over Annapurna from Pokhara

**Figure 2**

Campylobacter sandwiches sold here

Buses in Nepal are built, reasonably enough, to accommodate the Nepalese, who are congenitally small, and are further stunted by a life-time of malnutrition. Being 6ft. 4in. tall, the length of my femur exceeded the distance between the seats. This proved uncomfortable at first, and became excruciating later. The roof was correspondingly low, so that whenever we went over a pothole at speed, which was frequently, the entire company rose as one, but mine was the only head to strike the roof with any force. My travelling companions spoke little English, but were clearly sympathetic to my plight.

The neighbour on my right turned out to be a gaine (pronounced 'gynae', as in obstetrics), a professional beggar-musician who carried a 3-stringed fiddle crudely carved from a single block of wood. 'Gynaes' eke out a precarious living in rural Nepal by travelling from village to village like medieval troubadors. My 'gynae' friend, noting my interest in his fiddle and sensing business, played me a charming little Nepalese melody, accompanied by a pleasantly plaintive song. Encouraged by my response, he went into an encore which to my amazement was recognizable as 'Frere Jacques'. The tune was fairly accurate, but my God, the poor lyrics! After that I wouldn't have been surprised if he'd tackled Bach's Toccata and Fugue in D Minor, but he obviously realised he'd exhausted my supply of small change. He was an attractive little fellow, but I have to report with regret that he played a lot better than he smelt.

The Nepalese landscape resembles the pleats of a piano-accordion on a gigantic scale. The bus would grind its way laboriously up huge hillsides, negotiating 20 or 30 hairpin bends on the ascent, then teeter at the top of the pass before plunging down the opposite side with reckless abandon. The windows were small, but between the heads of the other passengers I caught occasional glimpses of Arcadian vistas—terraced fields of vivid green, stark stony hillsides, ricketty bridges over great gorges, small banana plantations, women pounding their washing on smooth rocks in the river, children leading large white bullocks, distant snowy peaks, and groups of animated girls, their gaudy saris sparkling in the sunshine.

Inside the bus, however, things were less idyllic. The atmosphere became steadily more oppressive, the discomfort in my cramped limbs increased, and about 4 hours out of Kathmandu I began seriously to regret drinking so much coffee for breakfast. Fortunately my need was not unique and eventually the bus stopped in a deserted spot and most of the passengers alighted. My 'gynae' friend made a gesture as though he was brushing crumbs from his lap, and made a noise which should have been 'Wee-wee', but sounded more like 'Ffff-fff'. Much more onomatopoeic, I thought. None of us attempted to hide behind rocks, though most of us did face away from the bus, but since some of the men were squatting with bare buttocks, it seemed debatable whether this was in fact the most discreet and socially acceptable orientation. The ladies assumed thoughtful expressions as they crouched demurely in a genteel group some 15 yards down

**Figure 3**

Temple at Bhaktapur with animal rights

**Figure 4**

Swayambhu Temple, Kathmandu
The all-seeing eyes of Buddha the road. I began to realize why Victorian lady travellers, experienced in the ways of the Orient, used to wear long skirts and no knickers. Nepalese ladies in general, being mainly Hindu or Buddhist, are delightfully free from the tiresome over-modesty which can be so dangerous to the male traveller in some Moslem countries. Throughout the journey, as they pushed their way in and out of the over-crowded bus, ladies of all ages squashed various parts of their anatomy into me with no sign of bashfulness other than a disarming giggle. My 'gynae' friend's wife was even less afflicted by the claims of modesty. She clearly suffered from a mobile source of cutaneous irritation and at one stage, following a particularly prolonged bout of scratching, she hoicked up the top part of her dress to expose her generous bosom, and proceeded to conduct a close inspection of the left nipple for the offending parasite. I tactfully averted my gaze, but my 'gynae' friend merely smiled and shook his head tolerantly.

In due course we reached a small market-town, where excited vendors pushed various small items of food through the windows in exchange for exceedingly grubby notes of small denomination. My gynae friend ate one boiled egg, a small quantity of cooked peas in a twist of paper, and about 5 peanuts. This was all he ate during the 10 hour journey, and his nutritional state suggested that this was his normal caloric intake for the day, if his audience had been generous.

As the hot afternoon wore on, my gynae friend had to turn his attention to his wife, who was leaning forward, clutching her head and moaning quietly. He was very sweet and supportive, cradling her in his arms and crooning a lullaby to her. As time went by the bus became even hotter, smellier and bumpier, and the moaning became more piteous. My own gastric quietude was already discombobulated by the universal Nepalese habit of noisy hawking and spitting, which was in no way inhibited by close confinement in a crowded bus. The ladies did have the grace to lean forward, lift their skirts and aim for the floor between their feet, but the men were less punctilious. They also added variety by blowing their noses out of the windows. My diaphragmatic self-control was just about adequate, provided I closed my eyes, clenched my teeth and breathed deeply until the noise abated. This was more than could be said for my 'gynae' friends' wife, who after a slow crescendo of moaning, leaned carefully over her husband's knee and vomited. Very little went on my shoe.

My earnest prayer for release from this hellish sardine-can was soon answered. The bus coughed, spluttered, groaned and stopped. Once in the fresh air, my relief gradually changed to concern, for we'd passed no garage since leaving Kathmandu 7 hours previously. There was, however, in the universal catch-phrase of the Indian sub-continent, 'No problem'. Rocks were wedged behind the wheels, tools miraculous-

*(continued on page 144)*

**Figure 4(b)**

### Foreign Correspondent. *(continued from page 140)*

ly appeared, several men disappeared under the engine, and after much banging and cursing, bits of bus innards were passed out to the 'pathologist' who read the entrails and predicted a delay of about 25 minutes. This prognosis proved remarkably accurate, and I later learned that one breakdown per journey is about par for the course.

When we eventually reached Pokhara it was dark. My gynae friend and his wife each shouldered huge bundles and cheerfully set off along the track to their own village, which was a further two days march away. As I alighted I was jostled by several small boys shouting 'Come with me sah, this my card, sah, very clean hotel sah, very cheap, follow me'. Feeling opulent, I plumped for the most expensive, at £3 per night. This turned out to be a civilized little guest house, which even had toilet paper. My sense of having endured an epic adventure began to diminish, and it was withered completely by the first guest I spoke to—a Danish girl who had just walked across Tibet.

## Figures and Tables

**Figure 1 f1:**
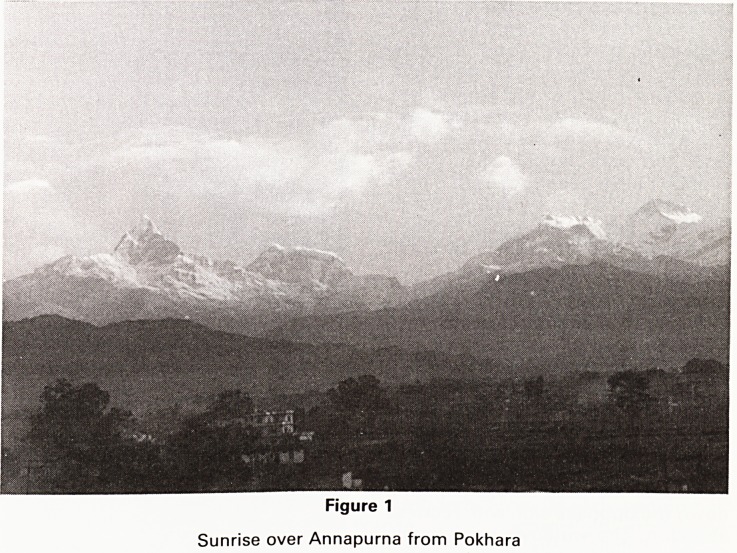


**Figure 2 f2:**
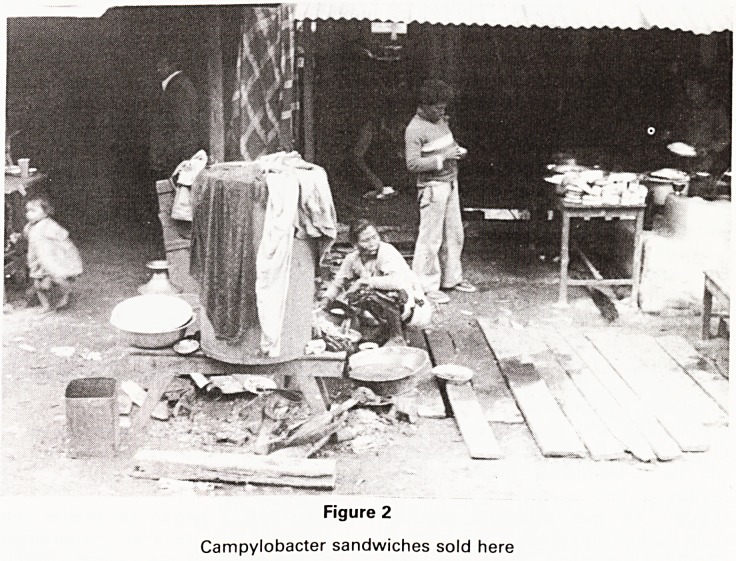


**Figure 3 f3:**
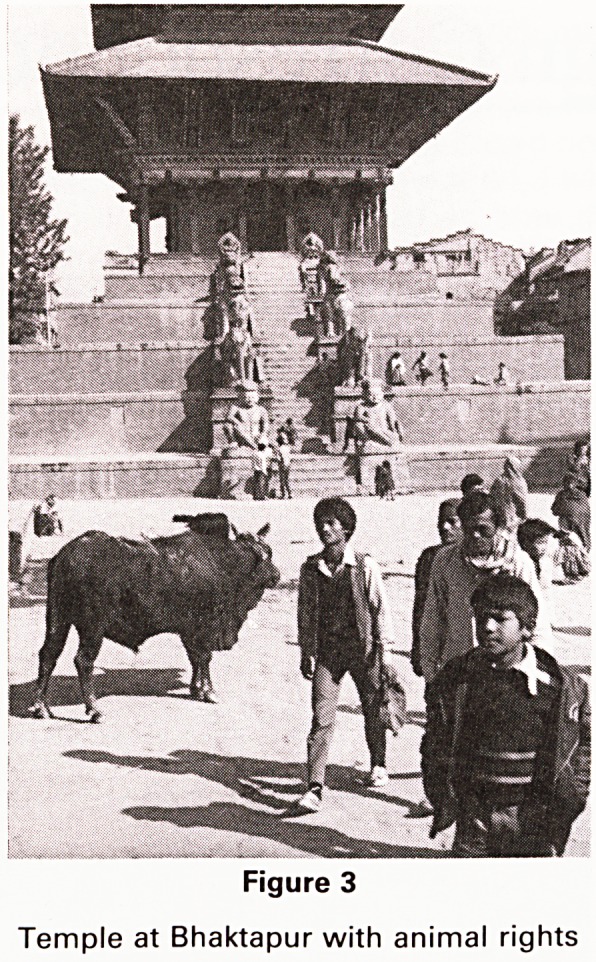


**Figure 4 f4:**